# Association of urogenital and intestinal parasitic infections with type 2 diabetes individuals: a comparative study

**DOI:** 10.1186/s12879-020-05629-9

**Published:** 2021-01-07

**Authors:** Babiker Saad Almugadam, Mihad Khaleil Ibrahim, Yinhui Liu, Shen-min Chen, Chun-hao Wang, Chen-yi Shao, Bao-wei Ren, Li Tang

**Affiliations:** 1 Department of Microecology, College of Basic Medical Sciences, Dalian Medical University, Dalian, Liaoning China; 2 Department of Microbiology, Faculty of Medical Laboratory Sciences, University of El Imam El Mahdi, Kosti, White Nile State Sudan; 3 Department of Medicine, Kosti Teaching Hospital, Kosti, White Nile State Sudan

**Keywords:** Helminthiasis, Intestinal parasitic infections, Parasitosis, Schistosomiasis, Type 2 diabetes mellitus

## Abstract

**Background:**

Globally, urogenital and intestinal parasitosis remain significant health challenges. They are associated with rising morbidity, death, and many harmful outcomes. A little is known concerning parasitosis and type 2 diabetes mellitus. Our study planned to investigate the urogenital and intestinal parasitic infections among type 2 diabetes patients compare to non-diabetic (Control) individuals and examine the intensity of helminthiasis in both groups.

**Methods:**

At Kosti Teaching Hospital (Sudan), 300 Urine and 300 stool samples have collected from 150 type 2 diabetes and 150 control individuals, along with the socio-demographic data using a structured questionnaire. The parasitic infections were examined by direct sedimentation technique for urine specimens. Whereas, for fecal samples, simple-direct saline, formal-ether concentration, Kato-Katz, and modified Ziehl–Neelsen techniques were used.

**Results:**

Out of 150 type 2 diabetes patients studied, 31 (20.6%) and 14 (9.3%) had intestinal parasitosis and urogenital schistosomiasis, respectively. Whereas, 16 (10.6%) and 8 (5.3%) of the control group were infected, respectively. Compared to the control group, the odds of testing positive for either urogenital schistosomiasis (AOR: 2.548, 95% CI: 0.836–7.761, *P* = 0.100) or intestinal parasitic diseases (AOR: 2.099, 95% CI: 0.973–4.531, *P* = 0.059) were greater in diabetic individuals. Likewise, the intensities of helminthiasis were much higher in the diabetic patients and positively correlated with the duration of illness. The rate of urogenital schistosomiasis was also significantly different among the disease duration subcategories.

**Conclusions:**

Our study has highlighted the relationship of type 2 diabetes with urogenital and intestinal parasitic infections and enhanced our knowledge about the frequency of particular urogenital and intestinal parasites as well as the intensity of helminths infection in type 2 diabetes compared to non-diabetic individuals, which are important for further studies.

**Supplementary Information:**

The online version contains supplementary material available at 10.1186/s12879-020-05629-9.

## Background

Urogenital and intestinal parasitic infections are groups of infectious diseases, still major public health concerns, particularly in tropical and subtropical rural areas of developing countries [1–5] since they have high morbidity and case fatality rate and endemic in many countries. As well, they have a wide distribution and related to immunity weakness, lack of health education, poverty, and inadequate hygiene [1, 3]. *A. lumbricoides*, *S. haematobium*, *T. trichiura*, hookworm, and *E. histolytica* comprise the majorities of the predominant causes of these illnesses [2, 4, 5]. Worldwide, schistosomiasis is responsible for 240 million infected people [6, 7]. In 2012, 250 million world population required preventive therapy against schistosomiasis [8]. On the other hand, the intestinal parasitic infections (IPIs) affect 3.5 billion people globally, and around 450 million persons had infected with intestinal parasites [3]. The pathology of parasitic infections generally depends on several factors, such as the immunological status of infected individuals. Clinically, intestinal and urogenital parasites are responsible for many disorders extending from slight discomfort to dangerous conditions such as growth retardation, malnutrition, and anemia [7, 9]. Every year, schistosomiasis is responsible for 24,067 to 200,000 deaths [10, 11] in addition to 1.496 million of years lived with disability (YLD) as well as multiples harms consequences globally [12–14]. Likewise, the intestinal parasitic diseases are accountable for 39 million disability-adjusted life years (DALYs) globally [15].

Type 2 diabetes mellitus (T2DM) is a chronic disorder characterized by persistent hyperglycemia. It has attracted much caution due to high incidence, mortality, and inescapable complications [16–20]. Globally, it represents approximately 90% of diabetes in adults. Also, the number of T2DM patients was rising over the last years, with more than 500 million prevalent in 2018, which will likely increase in the coming years, especially in countries with low-income [17]. Moreover, it has been linked to significant mortality and lower quality of life [18]. Besides the generalized immunity weakness and reduction in T-cell response [19], T2DM accompanied by humoral immunity disorders, innate immunity deregulation [20], impairment of multiple organs [18], and gut microbiota alterations [16, 17]. Thus, due to the effect of short and long-term consequences, type 2 diabetes mellitus individuals can be at higher risk for infections, and this susceptibility is gain increased under poorly controlled diabetes. Regarding all these findings, it is relevant to know concerning the association between T2DM and parasitic diseases. Accordingly, we conducted a matched case-control study to investigate the frequency of urogenital and intestinal parasitic infections among T2DM and non-diabetic (control) individuals.

## Methods

This was a case-control comparative study carried out at Kosti Teaching Hospital from thirteen January to 5 March 2019. Kosti Teaching Hospital is a referral hospital locates in Kosti city and provides health services to the most population of Kosti locality and its neighbor areas. Kosti locality is in the White Nile State of Sudan. The population of Kosti locality composes of a heterogeneous fusion of ethnic communities. The main workings include agriculture and free business. Kosti locality has plagued with severe deficiencies in the healthful source of water and healthcare facilities. Our study was approved by the Ethics Review Committee of Kosti Teaching Hospital and strictly adhered to the rules of the Declaration of Helsinki for the measures concerning human subjects. Written informed consent had also obtained from all participants after the full clearing up of the objectives and procedures of the study. Participant’s confidentiality and privacy have been protected by ensuring no names appeared on any part of the report. Supplementary Fig. 1 displays the study flow diagram.

### Study subjects

T2DM and non-diabetic individuals (Outpatients) attended to Kosti Teaching Hospital with clinical suspicion of urogenital and gastrointestinal tract infections and aged 21–80 years were included in this study. For positive cases, suitable treatment was prescribed by the staff of the hospital as well as applied if it is necessary. Diabetes subjects have recurred if they have been diagnosed with T2DM as based on the American Diabetes Association at least before 1 year. Participant’s exclusion criteria include antimicrobial use at least before 2 weeks, the recent history of malignancy or immune-suppressive diseases, pregnancy, history of chemotherapy or radiotherapy, alcohol adduction, previous history of gastrointestinal tract (GIT) surgery, and age <21 or >80 years. The researcher ensured the fitness of participants for this study. 150 T2DM patients, along with 150 control individuals (Convenience samples), were included. 125 of T2DM patients were under anti-diabetics drug therapy (Therapeutic subgroup: 29 Metformin, 46 Glimepiride, 13 Insulin, 9 Glibenclamide, 1 Gliclazide, 27 Metformin+Glimepiride), and 25 depended on diets control (Non-therapeutic subgroup). Based on diabetes duration, the diabetic group was also further divided into 1–6 years, 7–12 years, and 13–18 years subcategories.

### Research data and samples collection

A constructed pretested questionnaire was included to gather the required socio-demographic information includes age, gender, residence, and marital status. The water source was also gathered. Age-associated body-mass index (BMI) was computed by anthropometric calculation based on age, height, and weight of the participants. There was no significant difference (*r* = 0.041, *P* = 0.469) in the arithmetic mean of BMI between T2DM (23.8) and control (23.7) individuals. A researcher has clearly explained the questionnaire and guides the participants to fill the form.

Every participant has given two labeled (Different color) containers. They were also directed to collect a fresh fecal specimen in Screw capped bottle with broad mouths including various parts of the stool, besides approximately 20 ml of urine sample in another container [9].

### Parasitological examination

The collected samples were analyzed immediately. For the detection of urogenital and intestinal parasites, all the urine and stool specimens were prepared and examined in duplicate. Any sample with either parasitic eggs, trophozoite, cysts, or oocysts (For stool sample only) was considered positive. Urine specimens were processed by direct sedimentation technique, as described previously [9]. The whole slide was imaged via microscopy to detect the presence of parasitic forms such as eggs or trophozoites. The result of *S. haematobium* is expressed as egg/10 ml, and the infection intensities considered light or heavy when ova count 50 < eggs/10 ml or ≥ 50 eggs/10 ml, respectively [9, 21].

Initially, all stool samples had checked for the evidence of parasitic infection such as the color; consistency; and presence of blood, mucus, and parasitic segments or whole parasite. Subsequently, each sample was examined by a simple-direct saline method, formal-ether concentration technique, and modified Ziehl–Neelsen (ZN) method (for Cryptosporidium and Cyclospora spp) as described formerly [9]. Helminths infections intensities were also counted for helminths positive samples by Kato-Katz technique (Triplicate thick smears using standard 41.7 mg template) [22, 23], and the mean fecal egg counts (FEC) has estimated and stated as eggs per gram of feces (EPG). Based on the World Health Organization (WHO) guidelines [21], the intensity of *S. mansoni* infection had classified as light (1–99 EPG), moderate (100–399 EPG), or heavy (≥ 400 EPG).

10% of specimens in every batch of urine or stool samples were randomly selected and re-checked by an experienced technician blinded to the results of the first examination. In the case of dissimilarity, the discordant samples were re-examined to reach an agreement and obtain more accurate findings.

### Data analysis

The data were revised and subsequently entered in Microsoft Excel 2010. A double recheck was adopted to ensure data accuracy. The whole and comparative analysis of T2DM and control data were carried out using the statistical package for social sciences (SPSS) software, version 21.0 (IBM Corp., Armonk, N.Y., USA). The prevalence of parasitic infections had expressed as number and percentages, whereas BMI and helminths ova count as the arithmetic means. Moreover, the normality test was used to differentiate between the parametric and nonparametric numerical data. Afterward, a two-independent samples t-test (Parametric) and two- independent samples Mann-Whitney U test (Nonparametric) were used. Pearson Chi-Squared and Fishers Exact test (When there was at least one cell with an expected count of less than 5) were included in the statistical analysis of categorical data. Binary and multinomial logistic regression assessed the association between dependent (Urogenital schistosomiasis or IPIs) and independent variables. The odds ratios (unadjusted and adjusted) and 95% confidence interval (CI) were calculated to see the strength of the association. Furthermore, Pearson correlation was involved to test the force of the relationship between dependent and independent variables. R > 0 was considered a positive correlation, and < 0 as a negative correlation. A *p*-value of less than 0.05 has selected for a statistically significant difference.

## Results

Table 1 displays the investigated socio-demographic characteristics of the research participants. Gender (**χ**^**2**^ = 0.000, *P* = 1.000), residence (**χ**^**2**^ = 2.885, *P* = 0.089), and occupation (**χ**^**2**^ = 3.645, *P* = 0.456) were insignificantly different between the groups. Whereas, age (**χ**^**2**^ = 41.071, *P*<0.000), marital status (**χ**^**2**^ = 11.616, *P* = 0.001) and education level (**χ**^**2**^ = 17.725, *P* = 0.001) of participants were showed significant variation between T2DM and control individuals. In both groups, the majority of participants were married, belonged to urban areas, and have a source of purified water (Table 1).

**Table 1:** Socio-demographic characteristics of the study participants

Variable	Number	Study groups			
		Control: N (%)	T2DM: N (%)	χ^**2**^	***P*** value
Total of participants	300	150 (50)	150 (50)	**–**	**–**
Gender					
Male	150	75 (50)	75 (50)		
Female	150	75 (50)	75 (50)	0.000	1.000
Age					
21–40 Years	91	71 (78)	20 (22)		
41–60 Years	133	51 (38.3)	82 (61.7)	41.071	<0.000
61–80 Years	76	28 (36.8)	48 (63.2)		
Residence					
Urban	196	105 (53.6)	91 (46.4)		
Rural	104	45 (43.3)	59 (56.7)	2.885	0.089
Marital status					
Married	250	114 (45.6)	136 (54.4)		
Single	50	36 (72)	14 (28)	11.616	0.001
Water Source					
Purified river water	190	101 (53.2)	89 (46.8)		
Un purified river water	52	21 (40.4)	31 (59.6)	4.762	0.190
Boreholes water	36	20 (55.6)	16 (44.4)		
Hafir water	22	8 (36.4)	14 (63.6)		
Education level					
No formal	73	35 (47.9)	38 (52.1)		
Primary school	106	38 (35.8)	68 (64.2)	17.725	0.001
Secondary school	52	34 (65.4)	18 (34.6)		
University	69	43 (62.3)	26 (37.7)		
Occupation					
Unemployee	144	75 (52.1)	69 (47.9)		
Employee	63	31 (49.2)	32 (50.8)		
Business	51	28 (54.9)	23 (45.1)	3.645	0.456
Farmer	24	8 (33.3)	16 (66.7)		
Driver	18	8 (44.4)	10 (55.6)		

Statistical differences were evaluated using the Pearson Chi-squared test. Employees include the workers of both government and private institutions. Hafir is an artificially constructed large hole in the land for rainwater collection during the rainy season (Hafir water uses for drinking and other purposes). *N* Number, *T2DM* Type 2 diabetes mellitus, χ^2^: Chi-square

Out of the 150 T2DM persons examined, 31 (20.6%) had infected with at least one type of intestinal parasite as well as 14 (9.3%) has urogenital schistosomiasis. Whereas, 16 (10.6%) and 8 (5.3%) of the control group were infected by intestinal parasitosis and urogenital schistosomiasis, respectively. Compared to the control group, the adjusted odds of testing positive for either urogenital schistosomiasis (AOR: 2.548, 95% CI: 0.836–7.761, *P* = 0.100) or IPIs (AOR: 2.099, 95% CI: 0.973–4.531, *P* = 0.059) was at least two times higher in T2DM individuals (Table 2). Additionally, the infection rate of *S. haematobium* (Table 2) as well as *S. mansoni, H. nana, E. histolytica*, and Cryptosporidium spp. were higher in diabetic individuals compared to controls (Table 3). Three cases of multiple infections caused by *E. histolytica* and Cryptosporidium spp. were detected in the T2DM group (Table 3). In this study, there was a significant variation in the intensity of *S. haematobium* (*r* = 0.437, *P* = 0.040) and *S. mansoni* (g = 2.019, *P* = 0.033) infection between T2DM and control group. Moreover, all the infected individuals had shown a light *S. haematobium* infection (Table 4).

**Table 2:** Urogenital schistosomiasis and intestinal parasitic infections among the T2DM and control groups

Type of infection	Study groups	Frequency		Logistic regression analysis			
				Unadjusted		Adjusted	
		N (%)	***P*** value	OR (95% CI)	***P*** value	AOR (95% CI)	***P*** value
Urogenital schistosomiasis	Control (*N* = 150)	8 (5.3)		1		1	
	T2DM (*N* = 150)	14 (9.3)	0.184	1.827 (0.743–4.494)	0.189	2.548 (0.836–7.761)	0.100
Intestinal parasitic infections	Control (*N* = 150)	16 (10.6)		1		1	
	T2DM (*N* = 150)	31 (20.6)	0.017	2.182 (1.137–4.187)	0.019	2.099 (0.973–4.531)	0.059

*S. haematobium* (Urogenital schistosomiasis) as well as *S. mansoni, H. nana, *Cryptosporidium spp*, and E. histolytica* (Intestinal parasites) represented the detected parasites among both control and T2DM individuals. The adjusted variables were gender, age, residence, marital status, water source, education level, and occupation. *AOR* Adjusted odds ratio, *CI* Confidence interval, *OR* odds ratio (Unadjusted), *N* Number, *T2DM* Type 2 diabetes mellitus

**Table 3:** Frequency of intestinal parasites among the study groups

Parasite species	Prevalence of parasites: N (%)			
	Control (***N*** = 150)	T2DM (***N*** = 150)	OR (95%CI)	***P*** value
*S. mansoni*	3 (2)	5 (3.3)	1.690 (0.396–7.200)	0.723^f^
*H. nana*	4 (2.6)	6 (4)	1.521 (0.420–5.502)	0.520
Cryptosporidium spp	6 (4)	11 (7.3)	1.899 (0.684–5.276)	0.212
*E. histolytica*	3 (2)	6 (4)	2.042 (0.501–8.319)	0.501^f^
*Cryptosporidium spp* + *E. histolytica*	0 (0)	3 (2)	–	0.247^f^

Pearson chi-squared test and Fisher exact test^f^ were used in the statistical analysis. *CI* Confidence interval, *N *Number, *OR* odds ratio, *T2DM* Type 2 diabetes mellitus

**Table 4:** Helminths infection intensity

Parasite species	Helminths infection intensity							
	The arithmetic mean of helminths egg count: urine (eggs/10 ml) and fecal (FEC/gram)				N (%)			
					Light		Moderate	
	Control	T2DM	Effect size value	***P*** value	Control	T2DM	Control	T2DM
*S. haematobium*	6.3	11.5	0.437	0.040^a^	8 (100)	14 (100)	0 (0)	0 (0)
*S. mansoni*	29.2	81.5	2.019	0.033	3 (100)	4 (80)	0 (0)	1 (20)
*H. nana*	69.9	115.9	1.125	0.120	–	–	–	–

Two independent samples Mann-Whitney U^a^ and two independent samples t-test evaluated the statistical difference between the numerical data. *FEC* Fecal egg count, *N* Number, *T2DM* Type 2 diabetes mellitus. Effect size: r and Hedges’ g (g) value were calculated for two independent samples Mann-Whitney U test and two independent samples t-test, respectively

Comparatively, males and rural area residents have a higher rate of urogenital schistosomiasis and IPIs in both groups. Between the study groups, the positivity rate of these infections among gender, age, marital status, and residence was more in T2DM compared to controls, but it is not significant except for IPIs among females, 41–60 age (Years), and unmarried participants. IPIs were also significantly more in unemployed participants of T2DM compared to control groups. Moreover, the unpurified river water users have a high positivity rate of IPIs; whereas, 21–40 age (Years), unmarried, and hafir water users were showed a high frequency of urogenital schistosomiasis. In the control group, we found a high rate of urogenital schistosomiasis and IPIs among the individuals of no formal (11.4%) and primary school education level (15.7%), respectively. Whereas, in the diabetic group, the individuals of no formal and secondary school education level showed a high frequency of IPIs (26.3%) and urogenital schistosomiasis (16.6%), respectively (Table 5).

**Table 5:** Prevalence (positivity rate) of urogenital schistosomiasis and intestinal parasitic infections among the study groups

Variable	Urogenital schistosomiasis: N (%)				Intestinal parasitic infections: N (%)			
	Control(***N*** = 150)	T2DM(***N*** = 150)	OR (95%CI) for T2DMversus Control	***P*** value	Control(***N*** = 150)	T2DM(***N*** = 150)	OR (95%CI) for T2DMversus Control	***P*** value
Gender								
Male	6 (8)	8 (10.6)	1.373 (0.452–4.169)	0.575	11 (14.6)	17 (22.6)	1.705 (0.738–3.940)	0.209
Female	2 (2.6)	6 (8)	3.174 (0.620–16.261)	0.276^f^	5 (6.6)	14 (18.6)	3.213 (1.094–9.436)	0.027
*P* value	0.276^f^	0.575			0.113	0.545		
Age								
21–40 Years	5 (7)	4 (20)	3.300 (0.795–13.703)	0.103^f^	7 (9.8)	3 (15)	1.613 (0.377–6.909)	0.685 ^f^
41–60 Years	2 (3.9)	7 (8.5)	2.287 (0.456–11.465)	0.481^f^	4 (7.8)	18 (21.9)	3.305 (1.050–10.405)	0.033
61–80 Years	1 (3.5)	3 (6.2)	1.800 (0.178–18.187)	1.000^f^	5 (17.8)	10 (20.8)	1.211 (0.368–3.987)	0.753
*P* value	0.803^f^	0.196^f^			0.397^f^	0.884^f^		
Residence								
Urban	3 (2.8)	5 (5.4)	1.977 (0.459–8.510)	0.476^f^	9 (8.5)	13 (14.2)	1.778 (0.722–4.376)	0.206
Rural	5 (11.1)	9 (15.2)	1.440 (0.447–4.638)	0.540	7 (15.5)	18 (30.5)	2.383 (0.896–6.339)	0.077
*P* value	0.053^f^	0.045			0.250^f^	0.017		
Marital status								
Married	4 (3.5)	12 (8.8)	2.661 (0.834–8.492)	0.087	13 (11.4)	26 (19.1)	1.836 (0.895–3.767)	0.094
Single	4 (11.1)	2 (14.2)	1.333 (0.216–8.249)	1.000^f^	3 (8.3)	5 (35.7)	6.111 (1.222–30.572)	0.030^f^
*P* value	0.095^f^	0.622^f^			0.763^f^	0.167^f^		
Water Source								
Purified river water	4 (3.9)	5 (5.6)	1.443 (0.375–5.550)	0.736^f^	10 (9.9)	13 (14.6)	1.557 (0.646–3.748)	0.221
Un purified river water	2 (9.5)	5 (16.1)	1.827 (0.320–10.443)	0.687^f^	4 (19)	10 (32.2)	2.024 (0.538–7.608)	0.292
Boreholes water	0 (0)	1 (6.2)	–	0.444^f^	2 (10)	5 (31.2)	4.091 (0.674–24.829)	0.204^f^
Hafir water	2 (25)	3 (21.4)	0.818 (0.106–6.337)	1.000^f^	0 (0)	3 (21.4)	–	0.273^f^
*P* value	0.052^f^	0.089^f^			0.560^f^	0.109^f^		
Education level								
No formal	4 (11.4)	3 (7.8)	0.664 (0.138–3.203)	0.703^f^	4 (11.4)	10 (26.3)	2.768 (0.780–9.828)	0.107
Primary school	0 (0)	6 (8.8)	–	0.086^f^	6 (15.7)	12 (17.6)	1.143 (0.391–3.338)	0.807
Secondary school	3 (8.8)	3 (16.6)	2.067 (0.372–11.483)	0.405^f^	2 (5.8)	3 (16.6)	3.200 (0.483–21.211)	0.327^f^
University	1 (2.3)	2 (7.6)	3.500 (0.301–40.652)	0.552^f^	4 (9.3)	6 (23.1)	2.925 (0.739–11.571)	0.161^f^
*P* value	0.067^f^	0.711^f^			0.598^f^	0.683^f^		
Occupation								
Un employee	2 (2.6)	5 (7.2)	2.852 (0.535–15.206)	0.260^f^	7 (9.3)	15 (21.7)	2.698 (1.027–7.087)	0.039
Employee	2 (6.4)	2 (6.2)	0.967 (0.128–7.326)	1.000^f^	2 (6.4)	8 (25)	4.833 (0.936–24.946)	0.082^f^
Business	0 (0)	3 (13)	–	0.085^f^	5 (17.8)	4 (17.3)	0.968 (0.228–4.122)	1.000^f^
Farmer	1 (12.5)	3 (18.7)	1.615 (0.140–18.581)	1.000^f^	1 (12.5)	2 (12.5)	1.000 (0.077–13.016)	1.000^f^
Driver	3 (37.5)	1 (10)	0.185 (0.015–2.286)	0.275^f^	1 (12.5)	2 (20)	1.750 (0.129–23.703)	1.000^f^
*P* value	0.005^f^	0.499^f^			0.575^f^	0.904^f^		

Pearson chi-squared test and Fisher exact test^f^ assessed the differences at a 95% level of significance. Employees include the workers of both government and private institutions. Hafir is an artificially constructed large hole in the land for rainwater collection during the rainy season (Hafir water uses for drinking and other purposes). *S. haematobium (*Urogenital schistosomiasis)as well as *S. mansoni, H. nana, *Cryptosporidium spp*,* and *E. histolytica* (Intestinal parasites) represented the detected parasites among both control and T2DM individuals. *CI* Confidence interval, *N* Number, *OR* Odds ratio, *T2DM* Type 2 diabetes mellitus

**Fig. 1:**
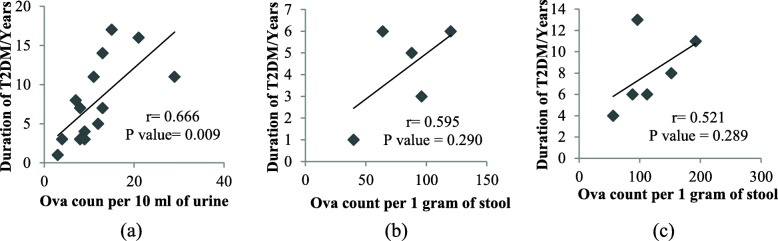
Correlation of helminths ova count with the duration of T2DM. **a**: *S.hematopium*, **b**: *S.mansoni,* **c**: *H.nana*, T2DM: Type 2 diabetes mellitus. Data assessed using Pearson correlation test

In the T2DM group, the prevalence rates of urogenital schistosomiasis and IPIs were nonsignificantly different between the diabetic subgroups, however, the frequency of IPIs was more in the non-therapeutic subgroup. Notably, the frequency of urogenital schistosomiasis was significantly different among the T2DM duration subcategories, which was 4.8%, 23.1%, and 15.8% among T2DM individuals with a disease duration of 1–6 years, 7-12 years, and 13–18 years, respectively (Table 6). Likewise, the intensity of helminthiasis had effected by the duration of T2DM. Indeed, the intensity of *S. haematobium* (R = 0.666, *P* = 0.009), *S. mansoni* (R = 0.595, *P* = 0.290), and *H. nana* (R = 0.521, *P* = 0.289) was positively correlated with the duration of T2DM (Fig. 1a-c).

**Table 6:** Link of parasitosis (positivity rate) with management policy and duration of T2DM

Variable		Urogenital schistosomiasis		Intestinal parasitic infections	
		N (%)	***P*** value	N (%)	***P*** value
T2DM subgroups	Therapeutic (*N* = 125)	12 (9.8)		25 (20)	
	Nontherapeutic (*N* = 25)	2 (8)	1.000^f^	6 (24)	0.652
Duration of T2DM	1–6 Years (*N* = 105)	5 (4.8)		21 (20)	
	7-12 Years (*N* = 26)	6 (23.1)	0.009^f^	6 (23.1)	0.950^f^
	13-18 Years (*N* = 19)	3 (15.8)		4 (21.1)	

The statitsical difference in the positivity rate of parasitic infections was assessed by the Pearson chi-square test and Fisher exact test^f^. *N* Number, *T2DM* Type 2 diabetes mellitus

## Discussion

Parasitosis influences many of the world’s population. Both urogenital schistosomiasis and IPIs have been linked to increased morbidity and mortality and serious complications. Intestinal parasitosis impairs the body’s metabolism, nutrition absorption, and gut ecosystem [24–26]. A complicated relationship exists between parasitosis and T2DM. The mechanisms of both suggest they influence each other. Previous studies showed that some helminths infection or its derived antigens reduce blood glucose and improve insulin sensitivity [27–31]. However, the exact mechanisms are not fully understood, this was explained by the anti-inflammatory effect of helminths since helminthiasis lower the circulatory pro-inflammatory cytokines and increase the anti-inflammatory cytokines [30, 31]. Helminths also lead to depletion of body energy sources resulting in weight loss and improved metabolic outcomes [27–29] as well as manipulate the gut microbiome that plays an essential role in blood glucose homeostasis [30, 32]. The current study findings showed that the proportion of parasitosis was much higher in diabetic individuals and the odds of testing positive for either urogenital schistosomiasis or intestinal parasitic diseases were more than two times higher in T2DM compared to control individuals. Several studies had documented similar findings for IPIs in T2DM [33–35] or diabetes mellitus (T1DM + T2DM) as general [35–38]. In contrast, some scholars [39, 40] found that diabetes mellitus (T1DM + T2DM) patients have a lower probability of intestinal parasitosis. Previously, Akinbo FO et al. [35] and Machado ER et al. [41] studies stated that the frequency of IPIs is lower in T1DM compared to T2DM individuals, which are of potential interest in the interpretation of the findings of Tangi FB et al. [39] and Nazligul Y et al. studies [40]. The variation between our findings and other studies outcomes may be attributed to differences in sample size; study population, area, and season; detected parasites; and diabetic management protocol, which are known confounding factors. In line with the Nazligul Y et al. study [40], our study found no significant difference in the rate of IPIs between T2DM subgroups. Interestingly, there was a significant variation in the frequency of urogenital schistosomiasis among the T2DM duration subcategories. Likewise, we found a positive correlation between helminths egg counts and T2DM duration. In these findings, the effect of age and other confounding factors are of potential importance. Similar to our study, Akinbo FO et al. study [35] found no significant variation in the frequency of IPIs among the duration of diabetes mellitus (T1DM + T2DM). In agreement with this study, Mohtashamipour M et al. study [33] found that the percentage of mixed infections was more in T2DM patients compared to control individuals. Tangi FB et al. study [39] also reported a high percentage of mixed infection in diabetes mellitus (T1DM + T2DM) than the control group. Taken together, these findings have underlined the link of T2DM with urogenital schistosomiasis and IPIs, which could be related to the generalized immunity weakness, gut microbiota changes, and the influence of short and long-term diabetes complications.

Regionally, the occurrence of urogenital and intestinal parasites influence by multiple elements including the environmental factors and geographic location, which can make a person at higher risk for particular parasites or indirectly affect the parasite survival, distribution, and transmission [1, 23, 35, 39]. In this study, *S. haematobium*, *S. mansoni*, *H. nana*, Cryptosporidium spp., and *E. histolytica* compromised the identified parasites, with considerably higher prevalences in diabetic individuals. Notably, those parasites are commonly detected in diabetic patients [33–41]. The previous studies were also reported a high frequency of *H. nana,* Cryptosporidium spp.*,* and *E. histolytica* among T2DM patients compared to the control group [33–35], which may be attributed to immunity weakness [19, 20], impairment of multiple organs, and other diabetic complications [17, 18].

Notably, the males and rural area residents have a higher rate of urogenital schistosomiasis and IPIs. This can be clarified, at least partially, by the fact that males are in more interaction with environments compared to females in this area. Moreover, the rural areas of Kosti locality suffer from infrastructures and health serves deficiency, in addition to the lack of the proper source of water supply, which could reflect the higher prevalence of diseases in a rural area compared to urban regions. Previously, several studies were stated a high positivity rate for urogenital schistosomiasis and IPIs in males [2, 3, 23]. Unlike our study, Tangi FB et al. study [39] found a high frequency of IPIs among females in both diabetes mellitus and control group. Likewise, the Akinbo FO et al. [35] and Nazligul Y et al. studies [40] of IPIs in diabetes mellitus (T1DM + T2DM) patients also reported a higher rate of IPIs among females than males. Dissimilar to our findings, Tangi FB et al. [39] study found a high frequency of IPIs in urban than rural areas residents. In this study, the frequency of urogenital schistosomiasis and IPIs among gender, age, marital status, and residence, was more in T2DM subjects compared to the control group; however, it is only significant for IPIs in females, 41–60 age (Years), and unmarried individuals. Akinbo FO et al. study [35] reported similar findings of the IPIs. This may strengthen the evidence of T2DM and parasitosis relationship. Furthermore, we found that the unpurified river water users have a high frequency of IPIs; whereas, hafir water users were shown a high rate of urogenital schistosomiasis. In our study, the frequency of urogenital schistosomiasis and IPIs among the control group was more in the individuals of no formal and primary school education level, respectively. Likewise, the participants of no formal and secondary school education level have a high rate of IPIs and urogenital schistosomiasis in the T2DM group, respectively. Akinbo FO et al. study [35] was reported a high rate of IPIs among the no formal educated diabetes mellitus (T1DM + T2DM) patients. Previously, the low education levels and unclean water were also linked to a rising probability of parasitosis [42]. Altogether, these findings highlight the impact of socio-demographic features in the frequency of urogenital schistosomiasis and intestinal parasitosis and suggest that the urbanization together with various improvements due to education and availability of a proper source of water supply could help to decline the prevalence of these infections.

In our study, the use of a single urine and stool sample together with lower diagnostic sensitivity methods and lack of the data of several socio-demographic features could affect the outcomes of the study. Because to have more accurate results, the use of multiple samples and highly sensitive diagnostic procedures such as the genetic techniques are necessary [42, 43]. Additionally, the incomplete matched design for the socio-demographic characteristics of the study participants such as age and education levels may also affect the study findings.

## Conclusions

In conclusion, our study indicated the association of T2DM with urogenital and intestinal parasitic infections and provided data about the frequency of particular urogenital and intestinal parasites as well as the intensity of helminths infection in type 2 diabetes compared to non-diabetic control individuals, which are important for further studies.

## Supplementary Information

**Additional file 1: Figure S1.** The study flow diagram.

## Data Availability

The data used in this study are available from the corresponding author on reasonable request.

## Abbreviations

BMI: Body mass index
CI: Confidence interval
DALYs: Disability-adjusted life years
EPG: Eggs per gram of feces
FEC: Fecal egg counts
GIT: Gastrointestinal tract
IPIs: Intestinal parasitic infections
T1DM: Type 1 diabetes mellitus
T2DM: Type 2 diabetes mellitus
WHO: World Health Organization
YLD: Years lived with disability
ZN: Ziehl–Neelsen
