# Supplementary figures and images for: Association of urogenital and intestinal parasitic infections with type 2 diabetes individuals: a comparative study
Source: BMC Infect Dis. 2021 Jan 7;21:20. doi: 10.1186/s12879-020-05629-9 (PMC7789604; doi:10.1186/s12879-020-05629-9)

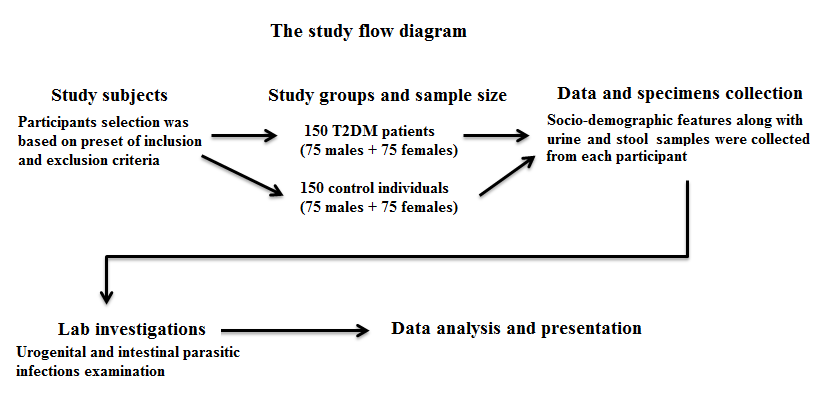


Supplementary Fig. 1The study flow diagram

Supplement: Supplementary file 1 — Additional file 1: Figure S1. The study flow diagram. [file 12879_2020_5629_MOESM1_ESM.docx]
